# Post-dural puncture headache in patients ≥ 50 years: a multicenter cohort study

**DOI:** 10.1016/j.bjane.2026.844821

**Published:** 2026-08-25

**Authors:** Scott Seadon, Andrew Walker, Abraham O. Fifen, Joanna J. Fifen

**Affiliations:** a University of Calgary, Cumming School of Medicine, Department of Anesthesia, Perioperative and Pain Medicine, Calgary, Alberta, Canada; b University of Calgary, Cumming School of Medicine, Department of Emergency Medicine, Calgary, Alberta, Canada; c University of Saskatchewan, Department of Public Health and Preventive Medicine, Saskatoon, Saskatchewan, Canada

*Dear Editor,*

Post-Dural Puncture Headache (PDPH) remains a recognized complication of spinal anesthesia, with needle design traditionally emphasized as a key modifiable risk factor.^1^ Atraumatic (pencil-point) needles are widely recommended due to their lower associated PDPH rates.^2^, ^3^, ^4^ However, PDPH appears to be uncommon in older adults, consistent with the well-established inverse relationship between age and PDPH risk.^1^^,^^5^ Consequently, two clinically relevant questions arise in this population: how frequently does clinically confirmed PDPH occur, and does needle type remain an important determinant of risk when the underlying event rate is already very low? Given the anticipated rarity of PDPH in older adults, this analysis was principally intended to describe event frequency and explore, rather than definitively test, any association between needle type and PDPH.

We conducted a retrospective multicenter cohort study of patients ≥ 50 years undergoing orthopedic surgery under spinal anesthesia. Our primary objective was to characterize the incidence of clinically confirmed PDPH in this older population, while secondarily exploring whether PDPH occurrence differed according to spinal needle type (22-gauge Quincke vs. 25-gauge Whitacre). Potentially eligible patients were identified retrospectively through institutional perioperative electronic medical records. Patients receiving spinal anesthesia with either a 22-gauge Quincke (cutting) or 25-gauge Whitacre (pencil-point) needle between May 2022 and February 2025 across four acute care hospitals were included. The primary outcome was clinically confirmed PDPH within three weeks of surgery, defined as a clinician-documented diagnosis of PDPH attributed to dural puncture rather than a non-specific postoperative headache. PDPH typically presented as a severe, positional headache accompanied by nausea, vomiting, tinnitus, photophobia, and neck stiffness, consistent with routine clinical documentation and assessment. The three-week follow-up window was selected to capture delayed presentations occurring after hospital discharge, including emergency department visits, anesthesiology consultation assessments, or epidural blood patch procedures. Secondary outcomes included documentation of any postoperative headache, recognizing that non-specific headache is common in the postoperative setting. Inclusion criteria included 22-gauge Quincke, or 25-gauge Whitacre needles used for spinal anesthesia. Exclusion criteria included use of other needle types or gauges, multiple needle attempts with different sizes, failed spinal anesthesia requiring conversion to general anesthesia and repeat surgeries as only the first procedure was considered for each patient. Because needle choice was not randomized and appeared to reflect anticipated technical difficulty, comparisons between needle types should be interpreted as exploratory and hypothesis-generating.

Incident rates with Clopper-Pearson 95% Confidence Intervals (95% CI) and absolute risk differences using 10,000 bootstrap replicates were calculated for documented headaches and PDPH. Relative effects of needle size on headaches and PDPH were assessed using binomial logistic regression and Firth penalized regression respectively and presented with Odds Ratios (OR) and 95% CI. We used a three-moment entropy balance weighting framework to balance demographic variable means, variances, and skewness between needle types. Variables included in the entropy model were age, sex, weight, height, Body Mass Index (BMI), American Society of Anesthesiologists’ (ASA) physical status, and procedure regressed on needle type using an Average Treatment effect in the Treated (ATT) estimand. The number of spinal needle insertion attempts was omitted from the weighting model as it represents a post-treatment variable that may lie on the causal pathway between needle type and our outcomes. We considered acceptable balance to be an absolute standardized mean difference < 0.1. Equivalent weighted binomial and Firth penalized regression analyses were completed that incorporated entropy weights. Robust sandwich standard errors were applied to the weighted binomial model. We considered p < 0.05 to be significant.

A total of 6,306 patients met inclusion criteria, including 418 in the Quincke group and 5,888 in the Whitacre group. There were marked baseline differences between groups. Patients receiving Quincke needles were older, had higher ASA physical status, and more frequently underwent procedures associated with technical difficulty (e.g., hip fracture and hemiarthroplasty) (Table 1). They also required a greater number of spinal needle insertion attempts. Together, these findings strongly suggest that needle choice was driven by anticipated anatomical and procedural challenges rather than random allocation, introducing substantial confounding. Consequently, this study should not be interpreted as a comparative effectiveness analysis and cannot establish equivalence between Quincke and Whitacre needles with respect to PDPH risk.

**Table 1:** Baseline and procedural characteristics by spinal needle type.

	25-gauge Whitacre needle patients (n = 5888)	22-gauge Quincke needle patients (n = 418)	p-value	ASD	ASD after entropy balancing
**Age, years, median [IQR]**	70.0 [63.0‒77.0]	77.0 [68.0‒84.3]	< 0.001	0.635	0.077
**Sex, female, n/total, n (%)**	3469/5888 (58.9)	252/418 (60.3)	0.582	0.028	0.012
**Weight, kg, median [IQR]**	80.7 [67.9‒94.3]	76.6 [62.1‒94.4]	0.009	0.085	0.040
**Height, m, median [IQR]**	1.65 [1.57‒1.73]	1.65 [1.57‒1.73]	0.850	0.023	0.004
**BMI, kg·m^-2^, median [IQR]**	29.1 [25.3‒33.5]	28.8 [24.7‒33.6]	0.266	0.029	0.012
**ASA physical status, n/total, n (%)**			< 0.001		
1	331/5861 (5.6)	6/414 (1.4)		0.186	0.001
2	3571/5861 (60.9)	156/414 (37.7)		0.470	0.022
3	1859/5861 (31.7)	230/414 (55.6)		0.525	0.016
≥ 4	100/5861 (1.7)	22/414 (5.3)		0.280	0.007
**Procedure**			<0.001		
Total knee arthroplasty	2783/5888 (47.3)	157/418 (37.6)		0.194	0.022
Total hip arthroplasty	1281/5888 (21.8)	53/418 (12.7)		0.242	0.008
Hip fracture	327/5888 (5.6)	85/418 (20.4)		0.451	0.033
Hemiarthroplasty	316/5888 (5.4)	68/418 (16.3)		0.356	0.005
Ankle fusion	190/5888 (3.2)	13/418 (3.1)		0.007	0.002
Partial knee replacement	125/5888 (2.1)	3/418 (0.7)		0.119	0.000
Other	866/5888 (14.7)	39/418 (9.3)		0.166	0.005
**Number of spinal needle insertion attempts, n/total, n (%)**			< 0.001		
1	4148/5674 (73.1)	165/411 (40.1)			
2	1026/5674 (18.1)	123/411 (29.9)			
3	367/5674 (6.5)	77/411 (18.7)			
4	81/5674 (1.4)	23/411 (5.6)			
≥ 5	52/5674 (0.9)	23/411 (5.6)			

ASD, Absolute Standardized Difference; IQR, Interquartile Range; BMI, Body Mass Index; ASA, American Society of Anesthesiologists.

Entropy balancing completed on pre-exposure demographic variables of age, sex, weight, height, BMI, ASA physical status, and procedure. Acceptable balance after entropy balancing was an ASD < 0.10.

*Maximum missing data was associated with patient height (5.4% in the 25-gauge group and 13.3% in the 22-gauge group). A complete-case analysis was used for all outcomes given the rarity of our post-dural puncture headache (n = 2). Such rarity can lead to separation issues and unstable estimates within a multiple imputation framework. Both patients experiencing post-dural puncture headache had complete baseline data and as such, use of a complete-case approach did not reduce statistical power.

Four-hundred and fifty-four total headaches were documented in 6,306 patients 7.2% (95% CI 6.6% to 7.9%). Postoperative headache of any type was documented in 10.0% (95% CI 7.3% to 13.3%) of patients in the Quincke group and 7.0% (95% CI 6.4% to 7.7%) in the Whitacre group. While the odds of postoperative headache were significantly higher in the Quincke group (Odds Ratio [OR = 1.48], 95% CI 1.05 to 2.05) on unadjusted analysis, this significant difference was no longer observed after adjustment for baseline imbalance using entropy balance weighting (OR = 0.98, 95% CI 0.71 to 1.37). This attenuation supports the interpretation that observed differences were attributable to patient and procedural factors rather than needle design per se. It also underscores the limitations of unadjusted comparisons in large observational datasets, particularly where treatment allocation is non-random.

Only two cases of PDPH were identified across the entire cohort of 6,306 patients 0.03% (95% CI 0.004% to 0.11%) with both occurring in the Whitacre group 0.03% (95% CI 0.004% to 0.12%). No cases were observed in patients receiving a Quincke needle 0.0% (95% CI 0.0% to 0.88%). Although the overall cohort was large, the very low number of PDPH events precluded meaningful comparative modeling or reliable estimation of between-group differences in PDPH risk. The absolute risk difference was -0.03% (95% CI -0.08 to 0.01%). Due to the zero-event count and large denominator imbalance between groups, Firth penalized regression resulted in an unstable sparse data estimated PDPH OR of 2.81 (95% CI 0.02 to 34.6, p = 0.553) in the Quincke group. The corresponding OR after entropy balancing was 3.50 (95% CI 0.02 to 52.6, p = 0.490). The extreme width of the 95% CI reflects large statistical imprecision rather than a true clinical increase in PDPH odds with a Quincke needle. Given the extremely low number of events, all comparative analyses were underpowered and should be interpreted as exploratory. Although the overall cohort was large, only two PDPH events were identified, precluding reliable estimation of between-group differences in PDPH risk. As such, no reliable inference can be made regarding the relative risk of PDPH between needle types. However, it is notable that even the upper bounds of estimated incidence remained very low in both groups, highlighting the rarity of clinically confirmed PDPH in this population.

These findings emphasize a key practical message: clinically confirmed PDPH was very rare in this patient cohort, with very few events observed in either needle group. This observation is consistent with existing literature demonstrating a strong inverse relationship between age and PDPH risk.^1^^,^^5^ Several mechanisms have been proposed, including decreased dural compliance, reduced cerebrospinal fluid leakage through puncture sites, and age-related changes in intracranial dynamics.^5^ In addition, altered pain perception and reporting in older adults may influence the clinical expression and recognition of PDPH.

In this context, the relative importance of needle design may be less pronounced than in younger populations. While atraumatic needles clearly reduce PDPH incidence in high-risk groups,^2^, ^3^, ^4^ the very low observed incidence of PDPH in this cohort suggests that any absolute difference attributable to needle type would likely be small, although this study was not powered to estimate that difference reliably. Consequently, other factors may reasonably guide spinal needle selection. Technical success and procedural feasibility are important considerations in elderly orthopedic patients, who often present with degenerative spinal changes, limited mobility, and reduced interspinous space. In technically challenging cases, clinicians may choose needle type based on perceived ease of use and procedural feasibility; however, the present study cannot determine whether one needle type improves procedural success, reduces insertion attempts, or minimizes patient discomfort.

From a pragmatic standpoint, our findings support a more individualized approach to needle selection in older adults. Rather than strict adherence to needle type based solely on PDPH prevention, clinicians may consider anatomical factors, anticipated difficulty, and operator experience when choosing equipment. This aligns with real-world practice patterns, as reflected in our data, where Quincke needles were preferentially used in more complex cases.

Several limitations should be acknowledged. First, the extremely low incidence of PDPH limits statistical power and precludes robust comparative conclusions. Although the overall cohort was large, only two PDPH events occurred, preventing reliable estimation of between-group differences in PDPH risk. Second, significant baseline imbalance reflects non-randomized needle selection and introduces substantial potential for confounding by indication despite statistical adjustment. Consequently, any comparison between needle types should be regarded as exploratory and hypothesis-generating rather than causal. Third, the retrospective design depends on clinical documentation, which may underestimate both headache frequency and diagnostic specificity. Misclassification between nonspecific postoperative headache and PDPH is possible. Finally, our findings may not be generalizable beyond older orthopedic populations, where baseline PDPH risk is already low.

In this multicenter retrospective cohort of orthopedic patients ≥ 50 years, clinically confirmed PDPH was exceedingly rare. Because only two PDPH events occurred, the study cannot support reliable comparative conclusions regarding needle type. These findings suggest that the absolute risk of PDPH in older orthopedic patients is low and that needle selection may reasonably incorporate technical considerations, patient anatomy, and operator judgment. Prospective studies with standardized PDPH ascertainment would be valuable to confirm these observations.

## Data availability statement

In accordance with the Alberta Health Services Data Disclosure Agreement, we are unable to provide or make the data available to a third party without prior written consent from Alberta Health Services, Calgary, Alberta, Canada. Custom-generated R code used for data analysis is available from the authors upon reasonable request, subject to institutional approval and applicable data-sharing agreements.

## Ethics approval

This study was approved by the University of Calgary Conjoint Health Research Ethics Board. Approval number: REB24-0612.

## Trial registration

Not applicable. Retrospective observational study.

## Declaration of AI and AI-assisted technologies

The authors declare that no Artificial Intelligence (AI) or AI-assisted technologies were used in the conception, data analysis, or writing of this manuscript.

## Authors' contributions

Scott Seadon: Study conception; Data acquisition; Manuscript drafting.

Andrew Walker: Statistical analysis; Data interpretation; Manuscript drafting and revision.

Abraham O. Fifen: Data interpretation; Critical manuscript revision.

Joanna J. Fifen: Study conception and design; Ethics oversight; Data interpretation; Manuscript drafting and revision; Final approval.

All authors meet ICMJE authorship criteria and approved the final manuscript.

## Funding

This research did not receive any specific grant from funding agencies in the public, commercial, or not-for profit sectors.

## Conflicts of interest

The authors declare no conflicts of interest.
